# Prolonged Dysfunction of Astrocytes and Activation of Microglia Accelerate Degeneration of Dopaminergic Neurons in the Rat Substantia Nigra and Block Compensation of Early Motor Dysfunction Induced by 6-OHDA

**DOI:** 10.1007/s12035-017-0529-z

**Published:** 2017-05-02

**Authors:** Katarzyna Kuter, Łukasz Olech, Urszula Głowacka

**Affiliations:** Institute of Pharmacology, Polish Academy of Sciences, 12 Smętna St., 31-343 Krakow, Poland

**Keywords:** Fluorocitrate, Astroglia, Microglia, Neuron–glia interaction, Behavioral compensation, Early Parkinson’s disease

## Abstract

Progressive degeneration of dopaminergic neurons in the substantia nigra (SN) is the underlying cause of Parkinson’s disease (PD). The disease in early stages is difficult to diagnose, because behavioral deficits are masked by compensatory processes. Astrocytic and microglial pathology precedes motor symptoms. Besides supportive functions of astrocytes in the brain, their role in PD is unrecognized. Prolonged dysfunction of astrocytes could increase the vulnerability of dopaminergic neurons and advance their degeneration during aging. The aim of our studies was to find out whether prolonged dysfunction of astrocytes in the SN is deleterious for neuronal functioning and if it influences their survival after toxic insult or changes the compensatory potential of the remaining neurons. In Wistar rat model, we induced activation, prolonged dysfunction, and death of astrocytes by chronic infusion of fluorocitrate (FC) into the SN, without causing dopaminergic neuron degeneration. Strongly enhanced dopamine turnover in the SN after 7 days of FC infusion was induced probably by microglia activated in response to astrocyte stress. The FC effect was reversible, and astrocyte pool was replenished 3 weeks after the end of infusion. Importantly, the prolonged astrocyte dysfunction and microglia activation accelerated degeneration of dopaminergic neurons induced by 6-hydroxydopamine and blocked the behavioral compensation normally observed after moderate neurodegeneration. Impaired astrocyte functioning, activation of microglia, diminishing compensatory capability of the dopaminergic system, and increasing neuronal vulnerability to external insults could be the underlying causes of PD. This animal model of prolonged astrocyte dysfunction can be useful for in vivo studies of glia–microglia–neuron interaction.

**Electronic supplementary material:**

The online version of this article (doi:10.1007/s12035-017-0529-z) contains supplementary material, which is available to authorized users.

## Introduction

The cause of Parkinson’s disease (PD) is the degeneration of dopaminergic neurons in the substantia nigra (SN) and diminished neurotransmission in its target structures, such as the striatum (STR). It is a slowly progressing disorder, leading to the motor disability, manifested by tremors, akinesia, bradykinesia, and many other accompanying peripheral and central symptoms. Interestingly, the first significant motor disturbances are not observed until the loss of dopaminergic neurons in the SN reaches as much as 70% which, in consequence, corresponds to at least 80% loss of dopamine (DA) in the STR [1]. The preclinical phase of this progressive degeneration, before onset of symptoms, is estimated to last 8–17 years [2].

Since this massive degeneration of dopaminergic system can go undetected for such a long time, the existence of compensatory mechanisms in early PD is an accepted fact [3–5]. Until a certain threshold of degeneration is reached, the remaining neurons and postsynaptic cells adapt to maintain near-normal functioning of the system [6–10]. The enhanced activity of the remaining dopaminergic neurons [11, 12] in the early stages of PD could make them additionally vulnerable to further insults. Furthermore, PD is strongly associated with aging, which attenuates compensatory mechanisms [13, 14]. Diminution of the brain’s natural protective and adaptive potential could be the underlying cause of dopaminergic neuron degeneration in PD [15]. Enhancing or supporting those natural mechanisms could be used as a potential PD treatment.

Astrocytes are one of the support systems for neuron functioning in the brain [16]. These ubiquitous cells interact with neurons at many levels. They not only are passive housekeepers but also actively participate in neuronal functioning. Astrocytes manage the production of neurotrophins (BDNF, GDNF, CNTF, NGF) [17], supply antioxidants (glutathione, ascorbate) to neurons, and regulate oxidative, osmotic, and ionic balance as well as dispose neuronal waste (ammonia) [18]. Astrocytic cells are responsible for blood–brain barrier formation, neurogenesis, and synaptogenesis. They cooperate with microglia in inflammatory response and can influence their state of activation [19–21]. Some astrocytes in the adult brain keep the role of progenitor cells [22]. They also form tripartite synapses and actively participate in neurotransmission by uptake and metabolism of the released neurotransmitters [23, 24]. Since they have neurotransmitter receptors on their surface, they can sense neuronal functioning and are even able to influence it by releasing gliotransmitters [24–27]. Importantly, astrocytes support neuronal energy metabolism through glucose uptake from the blood, producing and shuttling lactate, glutamine, and GABA to neurons [18, 28]. Furthermore, in the brain, these are mainly astrocytes that are capable of storing energy supply in the form of glycogen [29]. Although dopaminergic neurons in the SN have a particularly high metabolic rate [30–32], their cell bodies contain relatively small mitochondria mass, suggesting that they could be more dependent on energy supplies from astrocytes [18].

Astrocytes have been implicated in a broad spectrum of neurological, neurodevelopmental, and psychiatric disorders [33, 34], leading to the hypothesis that astrocyte pathology precedes neuronal damage in many such diseases [35]. In addition, normal aging, an important factor in PD, causes morphological alterations in astrocytes in the human SN, visible as increased glial fibrillary acidic protein (GFAP) expression and slight astrocytic activation [36]. There is evidence of astrocyte involvement in the pathologic process in PD. Astrocytes were shown to endocytose alpha-synuclein [37, 38]. In post-mortem brain samples, activated astrocytes with alpha-synuclein inclusions were found already at early disease stages and their number increased with its progression [39, 40]. Astrocytes with alpha-synuclein depositions activate microglial cells [41]. In addition, several of the proteins responsible for autosomal recessive forms of hereditary PD (Parkin, PINK-1, DJ-1) are mainly concentrated in astrocytes [41]. The astrocytic marker proteins — S100beta and GFAP — were found to be upregulated in the SN of PD patients [42]. Increased expression of proteins in astrocytes (GFAP, GMBF, galectin 1, sorcin A) was suggested to be a protective mechanism at early disease stages [43]. On the other hand, the levels of protective neurotrophins (NGF and BDNF) decrease in advanced PD [17]. All these data indicate an important role of not only neurons and activated microglia but also astrocytes in the pathogenesis of PD.

Since astrocytes are recognized as important supporters of neuronal functioning, we investigated whether their prolonged dysfunction and degeneration would influence neuronal cell death process, mimicking early PD. We were also interested to find out whether diminished astrocyte support would change the compensatory potential of the dopaminergic system to maintain its normal functioning after neurotoxin challenge. Compensatory potential is strongly dependent on the extent of neurodegeneration [6, 44]. There is a threshold of damage that cannot be overcome by remaining cells. Therefore, we performed our experiments in a rat model of medium-size lesion induced by selective anti-dopaminergic toxin 6-hydroxydopamine (6-OHDA). This model enabled us to study both transient motor deficit and its recovery, proving degeneration and the compensatory capacity of the remaining system.

Previous in vivo studies focused on acute metabolic dysfunction of astrocytes after fluorocitrate (FC) injection which was totally reversible after 48 h [45–47]. The genetic modifications in animals depleted glia usually in other than SN brain regions [48–52]. There is no direct evidence in literature that prolonged dysfunction of astrocytes influences dopaminergic neurons in the SN in vivo. In order to induce a chronic state of astrocyte dysfunction and investigate its long-term effects, we slowly infused a low dose of FC into the SN using osmotic minipumps for 7 days. Our studies showed that prolonged inhibition of astrocyte function and their death, as well as concurrent microglia activation, stressed dopaminergic neurons but did not kill them, although accelerated their degeneration induced by the toxin 6-OHDA. Furthermore, FC and degeneration of astrocytes temporarily disturbed locomotor behavior of animals. The SN devoid of astrocytic support showed a strong activation of microglia and enhanced dopamine turnover. Importantly, FC treatment blocked the compensatory potential of dopaminergic system to counteract neuronal degeneration induced by 6-OHDA.

This study shows the important role of astrocytes in early degeneration of nigrostriatal neurons and in the processes responsible for functional compensation of small dopaminergic deficits.

## Materials and Methods

### Animals and Stereotaxic Operations

Three-month-old male Wistar HAN rats (Charles Rivers, Germany) were kept under 12 h dark/light cycle (light from 06:00 to 18:00), with free access to food and water.

Stereotaxic brain operations were performed according to Kuter et al. [53], with modifications, under ketamine and xylazine anesthesia (65–50 and 10–3 mg/kg *im*, Biowet, Puławy, Poland). Desipramine (30 mg/kg *ip*, Sigma-Aldrich, Germany) was administered 30 min before lesioning to protect the noradrenergic terminals. To induce degeneration of dopaminergic neurons, the animals were stereotaxically, bilaterally injected with 6-OHDA HBr (3 μg base/3 μl per side), and dissolved in 0.2% ascorbic acid (both from Sigma-Aldrich, Germany) into the passing fibers of the medial forebrain bundle (MFB), at the following coordinates: AP 1.4 mm, L ±1.6 mm, and V 8.7 mm from bregma, according to [54]. Control, sham-operated rats received solvent in the same way. The injection cannula was left in place for 2 min for full absorption of the solution. Additionally, in the same animals, stainless steel cannulas were bilaterally and permanently implanted in the SN *pars compacta* (SNc) (coordinates: AP 4.9 mm, L ±1.8 mm, V 8.3 mm from bregma, according to [54]) and connected by a catheter to osmotic minipumps (1007D, ALZET, Charles-Rivers, Germany), implanted under the skin on the neck that administered fluorocitrate (FC, 2 nmol/day, Sigma-Aldrich, Germany) for 7 days, at a continuous rate 0.5 μl/h, to induce astrocyte dysfunction. On the 7th day after operation, the rats were anesthetized again and osmotic minipumps were explanted, catheters sealed, and skin closed again. Respective control animals had cannulas implanted with sealed catheters. FC was prepared according to Paulsen et al. [45]. To avoid infections, the rats received an antibiotic (100 μl/100 g, *ip*, Lincospectin, Pharmacia, Belgium) on the day of operation and 24 h afterwards. Body weight of animals was monitored during the whole experiment.

### Behavioral Analysis Using Automated Actimeters

Rat locomotor activity (path length, locomotion, and resting times) and rearings (total, free, and supported number, duration) were measured at different time-points after operation (3, 4, 5, 6, 26, 27 days) using computerized actimeters (ACTIFRAME-SYSTEM, GERB Elektronik GmbH, Berlin; Germany, with ARNO software) as described before in detail [53, 55]. Animals were placed in the cages individually with free access to food and water at 14:00 h, and their behavior was analyzed until 6:00 in the morning, consisting of both light (4 h) and dark phase (12 h) of the day. Each analysis session included animals from all treatment groups, from the same post-operation day. If the same animal was tested twice, the observations were at least 3 weeks apart.

### HPLC-EC Analysis of DA, Its Metabolites, and Turnover Rates

Rats were decapitated on 7th or 28th day after operation. The left STR and SN were immediately dissected and frozen on dry ice. Tissue was kept at –80 °C until further analysis. The levels of DA and its metabolites — 3,4-dihydroxyphenylacetic acid (DOPAC), 3-methoxytyramine (3-MT), and homovanillic acid (HVA) — as well as serotonin (5-HT) and its metabolite 5-hydroxyindoleacetic acid (5-HIAA) were assessed using an HPLC method with electrochemical detection as described previously [56]. Briefly, tissue samples were homogenized in 0.1 M perchloric acid containing 0.05 mM ascorbic acid and injected into the HPLC system (column Hypersil Gold C18, 100 × 3.0 mm, 3 μm, Thermo Scientific, UK) equipped with electrochemical detector analytic cell 5010 Coulochem III (ESA, Inc., USA). The mobile phase was composed of 50 mM NaH_2_PO_4_ × 2H_2_O; 40 mM citric acid; 0.25 mM 1-octanesulfonic acid sodium salt; 0.25 mM EDTA; 1.3% acetonitrile; and 2.4% methanol. The applied potential was E1 = −175 mV and E2 = +350 mV. The data were quantified using the area under peaks and external standards with Chromeleon software (Dionex, Germany). The turnover rates were calculated as metabolites to neurotransmitter ratios.

### Immunohistochemistry, Stereology, and Densitometry

#### Immunohistochemistry

After decapitation, the right brain hemispheres were rapidly removed, post fixed in cold 4% paraformaldehyde, and cryoprotected in sucrose solution. The brains were then cut on a freezing microtome into 30 μm frontal sections (AP −4.4 to 6.6 mm from bregma according to [54] for SNc–ventral tegmental area (VTA)) according to the stereological rules and stained as described before [57]. Free-floating sections were incubated in primary antibodies (anti-tyrosine hydroxylase (TH; AB_2201526); anti-GFAP (AB_2109645), both from Chemicon Int., USA; anti-Iba1 (AB_839504; WAKO, Japan)). For anti-S100 (AB_306716; Abcam, UK) staining heat-induced antigen retrieval in 10 mM citrate buffer pH 6.0 was performed. After incubation with secondary antibodies (anti-mouse (AB_2313571) or anti-rabbit (AB_2313606) biotinylated, Vector Laboratories, UK), sections were processed using an ABC-Peroxidase Kit (Vector Laboratories, UK) and 3,3′-diaminobenzidine as a chromogen. Subsequently, sections containing SNc–VTA stained for TH were counterstained with 1% cresyl violet (CV) with Nissl method. All sections were cover-slipped in a Permount medium (Fisher Scientific, USA).

#### Stereology

TH^+^ and/or CV^+^ neurons and S100^+^ astrocytes were counted stereologically in the SNc and VTA as described previously [58]. Stereological counting was performed using a light microscope (Leica, Denmark) controlled by a newCAST (Visiopharm, Denmark) software. The analyzed regions were outlined under lower magnification (×5), and their areas were estimated. The number of stained cells was calculated under ×63 magnification using a randomized meander sampling and the optical dissector methods.

#### Densitometry

The intensity of S100 staining in SNc was estimated on high precision scans of tissue sections (Scanner Epson Perfection V750 Pro, Seiko Epson Corporation, Japan) using Multi Gauge software (Fujifilm Holdings Corporation, Japan). Regions of interest were outlined, and mean quant level per area (QL/pixel^2^) was quantified from 9 to 12 sections per animal.

### Western Blot

Frozen tissue samples were lysed by sonication (21.5 μl lysis buffer/mg of tissue) in RIPA buffer (50 mM Tris; 150 mM NaCl; 1% NP-40; 0.1% SDS; 0.5% sodium deoxycholate; pH 7.4) supplemented with protease and phosphatase inhibitors. Protein concentration in supernatants was determined using Pierce™ BCA Protein Assay Kit (Thermo Scientific, USA). Protein samples were mixed with loading buffer, heat denaturized, and resolved by SDS–PAGE. Transfer to 0.2 μm PVDF membranes (Roche Diagnostics, Germany) was performed with semi-dry, discontinuous buffer system in Trans-Blot® Turbo™ (Bio-Rad, USA). Blots were probed with primary antibodies against ALDH1L1 (AB_10712968; Abcam, UK), GFAP (AB_2109645, Chemicon International Inc., USA), Iba1 (AB_839504; WAKO, Japan), S100beta (AB_2184554; Santa Cruz Biotechnology, USA), and β-actin antibodies (AB_626632; Santa Cruz Biotechnology, USA). Secondary anti-rabbit (Cell Signaling Technology, USA) and anti-mouse/rabbit (Roche Diagnostics, Germany) antibodies conjugated with horseradish peroxidase were used. Detection was done using chemiluminescence solution (0.1 M Tris; 5.3 mM H_2_O_2_; 1.25 mM luminol; 2.0 M 4-iodophenylboronic acid) [59]. After immunodetection, membranes were stained with Coomassie blue and total protein staining in lane was used as a loading control for each sample as described earlier [60]. Analysis was performed in duplicate or triplicate on each animal.

### Statistical Analysis

Results are presented as the mean ± standard error of the mean (SEM). The statistical analysis of results was performed using STATISTICA 10.0 software (StatSoft Inc., USA). *P* ≤ 0.05 was considered as statistically significant, and 0.1 ≥ *p* > 0.05 were considered as trends.

Analyses were done by a three way factorial ANOVA with the Fisher least significant difference (LSD) post hoc test or two-way ANOVA with the LSD post hoc test and *t* test for comparison of groups in time. A repeated measures ANOVA test was used for motor behavior analysis.

## Results

### Disturbed Motor Behavior Induced by a Medium-Size Dopaminergic System Lesion Compensates with Time but Not After FC Infusion

Several parameters of rat motor behavior were quantified: walking path length, number of rearings (all, free, supported), and time spent on locomotion, resting, and rearing. By extending the test duration and including both light and dark phase of the day, we increased the sensitivity of analysis. Crucial time-points after operation were chosen for behavioral analysis. The 4th day represented the time when active degeneration of dopaminergic cells and motor deficit were observed in the previous studies [53]. The 6th day showed the end of FC infusion and its cumulative effect. The 27th day was the time at which degeneration was fully accomplished, and behavioral deficit was already functionally compensated.

**Fig. 1:**
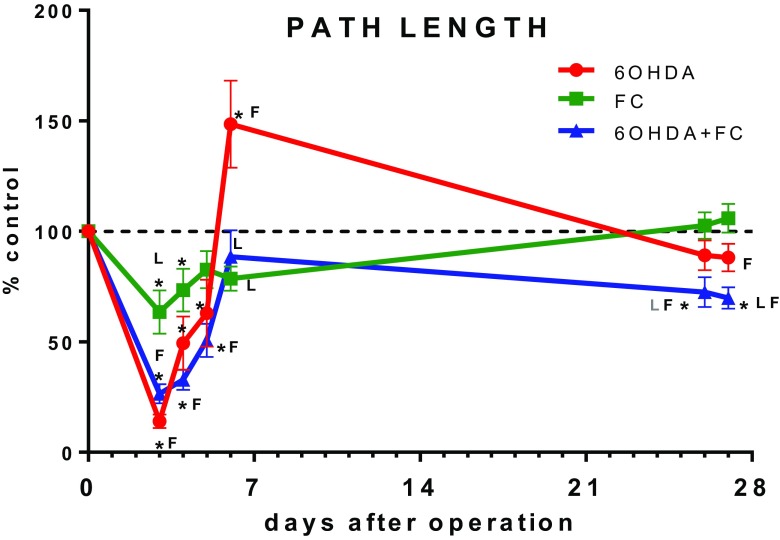
Path length analysis throughout the whole duration of the experiment. Data are presented as the mean values ± SEM in percent of sham + solvent controls from path length sum of 16 h analysis period at a given time-point after operation. Two-way ANOVA and the LSD post hoc test and *p* ≤ 0.05 were used for analysis within each time-point. Each group consisted of 5–15 animals. * vs sham + solvent; *L* vs 6-OHDA lesion + solvent; *F* vs FC + solvent

**Fig. 2:**
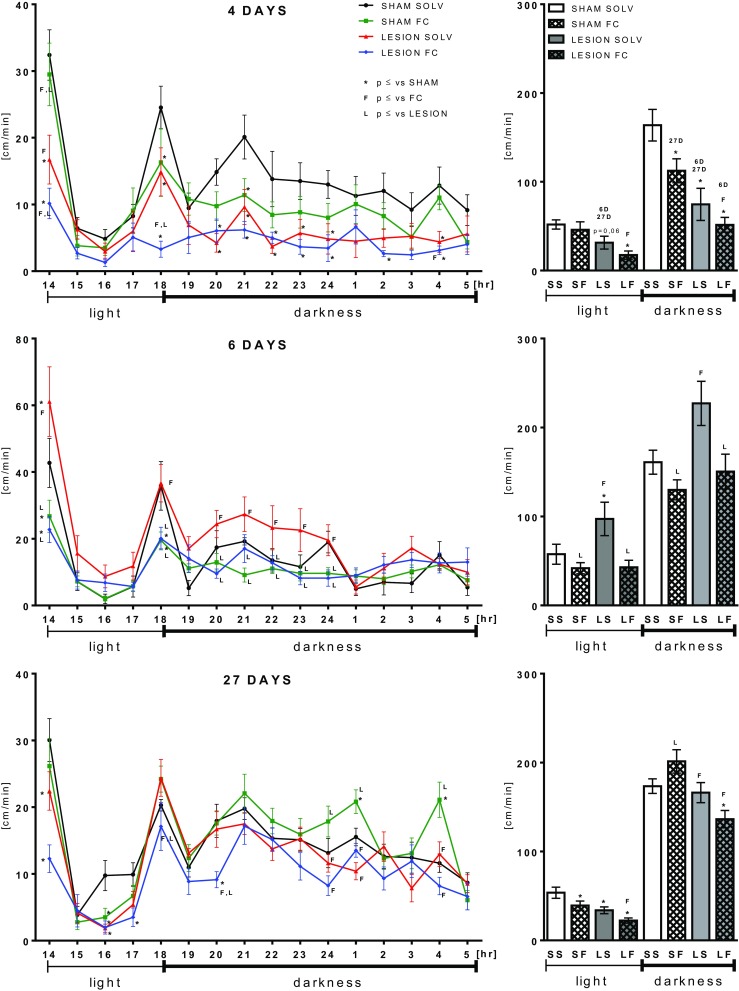
Locomotor activity shown as path length measured on days 4, 6, and 27 presenting the activity time line over 16 h of analysis (*left panel*) and as the total values in light and dark phases, separately (*right panel*). Data are presented as the mean values ± SEM of the activity summed from 60 min interval or as the sum from light or dark phases. Repeated measures ANOVA and the LSD post hoc test and *p* ≤ 0.05 were used. Each group consisted of 5–15 animals. * vs sham + solvent; *L* vs 6-OHDA lesion + solvent; *F* vs FC + solvent; *6D* vs 6-day time-point; *27D* vs 27-day time-point

We observed motor dysfunction in animals treated with 6-OHDA 4 days post lesion, manifested by the decreased walking path length (Figs. 1 and 2, Table 1); locomotion time; total, supported, and free rearings; and increased resting times (Table 1). The significant changes were more easily detected either during the 1st hour of analysis (exploratory activity) or in the dark phase comprising most of the nocturnal animal activities (Fig. 2). The most of behavioral deficits were diminished already after 6 days and even overcompensation occurred, manifested by higher values for walking path length, locomotion time, and free rearings. Interestingly, on the 27th day, no significant motor deficits were detected anymore in the 6-OHDA-treated animals vs sham-operated controls, indicating functional adaptation.

**Table 1:** Behavioral analysis of rat locomotor activity (path length, locomotion, and resting times) and rearings (total, free, and supported number, duration) on days 4, 6, and 27 shown as the total values from both light and dark phases of the day

		Path length (cm)	Locomotion time (% of time)	Rearing free (% of time)	Rearing time (s)	Rearing total (number)	Rearing side (number)	Rearing free (number)
4 days	SS	216 ± 21	12.7 ± 1.4	5949 ± 715	113 ± 6	12.5 ± 1.1	5.4 ± 0.6	7.1 ± 0.7
	SF	158 ± 21*	8.8 ± 1.4*	7643 ± 1244	98 ± 15	8.4 ± 1.6*	4.2 ± 0.7	4.2 ± 1.0*
	LS	107 ± 26*	5.3 ± 1.6*^F^	11844 ± 1875*^F^	89 ± 24	3.7 ± 1.5*^F^	2.5 ± 1.1*	3.2 ± 1.3*
	LF	71 ± 10*^F^	2.8 ± 0.5*^F^	10893 ± 899*^F^	38 ± 5*^LF^	2.0 ± 0.9*^F^	1.8 ± 0.8*^F^	0.2 ± 0.1*^LF^
6 days	SS	219 ± 21	12.4 ± 1.6	7221 ± 1132	104 ± 16	13.9 ± 0.8	6.2 ± 0.8	7.7 ± 0.7
	SF	172 ± 12^L^	8.9 ± 0.8^L^	5728 ± 644	128 ± 14	8.5 ± 0.8*^L^	4.0 ± 0.4*	4.5 ± 0.7*
	LS	324 ± 43*^F^	19.0 ± 3.2*^F^	4635 ± 636*^F^	119 ± 18	15.6 ± 2.3^F^	5.4 ± 0.8^F^	9.3 ± 1.2^F^
	LF	193 ± 26^L^	10.6 ± 1.9^L^	6040 ± 1231^L^	151 ± 26	7.6 ± 1.2*^L^	3.3 ± 0.5*^L^	4.3 ± 0.7*^L^
27 days	SS	227 ± 12	12.0 ± 0.9	4133 ± 534	132 ± 7	12.3 ± 0.8	6.0 ± 0.5	6.3 ± 0.4
	SF	241 ± 15	13.0 ± 0.9	4989 ± 744	117 ± 7	13.6 ± 0.7	6.5 ± 0.3	7.1 ± 0.7
	LS	200 ± 14^F^	10.1 ± 1.2^F^	3371 ± 315^F^	124 ± 8	11.5 ± 1.7	5.7 ± 0.9	5.8 ± 0.9
	LF	159 ± 11*^LF^	8.0 ± 0.7*^F^	7604 ± 1223*^F^	182 ± 22*^LF^	8.5 ± 0.9*^LF^	4.8 ± 0.6^F^	3.7 ± 0.4*^LF^

Data presented as the mean values ± SEM. Two-way ANOVA and the LSD post hoc test were used, and Student’s *t* test was applied to compare time effect between groups with the same treatment. Each group consisted of 5–15 animals

*p ≤ 0.05 vs sham + solvent; ^L^ *p* ≤ 0.05 vs 6-OHDA lesion + solvent; ^F^ *p* ≤ 0.05 vs FC + solvent

The chronic treatment with FC caused also a behavioral deficit manifested by a significant decrease in the walking path length, locomotion time, number of total and free rearings, and an increase in the resting time (Figs. 1 and 2, Table 1). The severity of motor deficit induced by FC was much smaller than that caused by the selective anti-dopaminergic toxin. Interestingly, after 6 days, at the end of FC treatment, the significant deficit was observed in the total rearing, while in other parameters, there were only some tendencies towards the decreased motor behavior. No overcompensation was visible. After 27 days, all parameters were normalized.

The combined treatment with 6-OHDA and FC induced behavioral deficits of the same magnitude as after 6-OHDA alone after 4 days (Figs. 1 and 2, Table 1). Interestingly, after 6 days, some parameters briefly returned to control levels (path length, locomotion, resting, and rearing times) but not the number of rearings. In contrast to 6-OHDA treatment alone, no overcompensation occurred. Prolonged motor deficits were still observed even after 27 days in all parameters and were significantly different from the effect of 6-OHDA or FC given separately.

### Loss of Phenotype and Accelerated Degeneration of Dopaminergic Neurons in the SN Due to FC Infusion

**Fig. 3:**
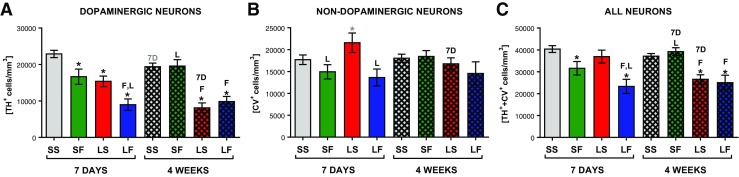
Stereological counting of dopaminergic (*TH* ^*+*^ */CV* ^*+*^) (**a**), non-dopaminergic (*TH* ^−^ */CV* ^+^) (**b**), and all neuron (sum of dopaminergic and non-dopaminergic) (**c**) densities in the SNc. Data are presented as the mean values ± SEM. Two-way ANOVA and the LSD post hoc test were used, and Student’s *t* test was applied to compare time effect between groups with the same treatment. * vs sham + solvent; *L* vs 6-OHDA lesion + solvent; *F* vs FC + solvent; *7D* vs 7-day time-point; *marks* in *black* indicate *p* ≤ 0.05 and *marks* in *gray* 0.1 ≥ *p* > 0.05. Each group consisted of 5–9 animals

After 6-OHDA injection to the MFB, stereological counting of neurons in the SNc revealed a progressing decline in the density of dopaminergic (TH^+^/CV^+^) neurons (by 32.8 and 64.6% of control, 1 and 4 weeks post lesion, respectively, *F*(2, 13) = 11.45, *p* = 0.0013) (Fig. 3a). Like in our previous studies [53] showing the loss of TH phenotype before actual degeneration of neurons, we observed a tendency towards the increase in non-DA neuron density (TH^−^/CV^+^) (Fig. 3b). There was no significant change in the pool of all neurons (dopaminergic TH^+^/CV^+^ + non-dopaminergic TH^−^/CV^+^) (Fig. 3c) after 1 week, and actual neuronal degeneration was visible only after 4 weeks with an overall decrease by 35%, indicating progressiveness of the degeneration after a single 6-OHDA injection into the MFB.

After 7 days of FC administration alone, we observed significantly decreased density of dopaminergic neurons and all neurons in the SNc to 72.7 and 78.2% of controls, respectively (Fig. 3a, c). Parallel lack of a significant decrease in non-DA neuron density after 7 days and lack of its decreases after 4 weeks suggested that the observed changes were due to the transient decrease in phenotype of TH^+^/CV^+^ neurons (Fig. 3b).

Concomitant 6-OHDA and FC treatment accelerated the degeneration of dopaminergic neurons but did not enhance it. Full degeneration was observed already after 1 week (decrease in TH^+^/CV^+^ neuron density by 39.2%) without further significant progression after 4 weeks (decrease by 42.9%) (Fig. 3a). A small decrease in density of non-DA neurons in the SNc was visible but was not statistically significant; hence, we cannot strongly conclude whether the astrocyte stress caused degeneration selectively to TH^+^/CV^+^ neurons, but it is plausible that DA neurons were mostly affected (Fig. 3b). Importantly, the size of degeneration after combined treatment was the same as the effect of 6-OHDA alone.

Similar effects were observed in the VTA region (data not shown).

### Prolonged FC Infusion Induces Astrocyte Death in the SNc

The tissue sections from the SN were stained with anti-GFAP or anti-S100 antibodies to visualize the expression of astrocytic markers. While GFAP was localized mostly in the ramifications, S100 was found predominantly in cell bodies. Interestingly, comparison of different control brain regions stained for GFAP or S100 revealed that a smaller number of glial cells were visible in the SNc than in the surrounding areas or the hippocampus and cortex. Also, morphology of astrocytes in the SNc was different, with fewer ramifications and they seemed smaller, indicating that astrocytes located in this dopaminergic structure were a distinct subpopulation (for comparison see refs. [61, 62]).

**Fig. 4:**
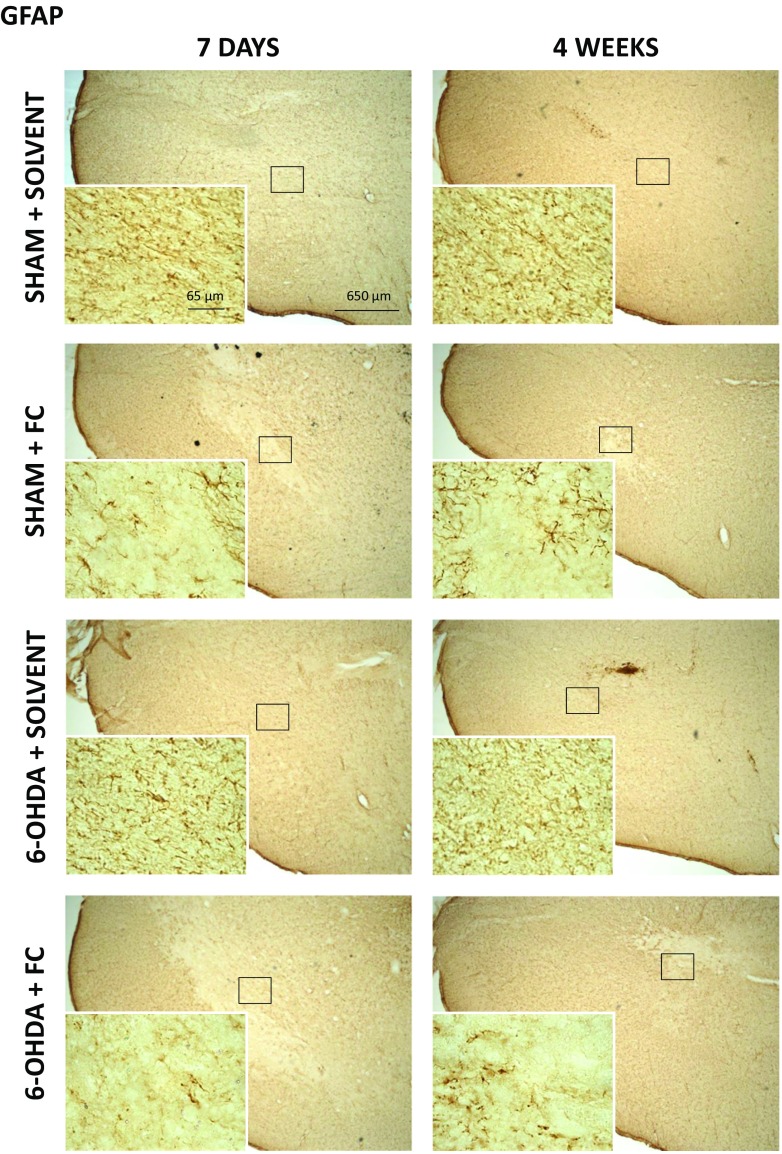
A representative immunohistochemical staining for GFAP^+^ astrocytes in the SN tissue sections under ×5 and ×63 magnifications. Diminished staining is visible in the region of SN after 7 days of FC infusion, and this effect is partially reversed after 4 weeks. Astrocyte activation is observed at the edges of the affected region

**Fig. 5:**
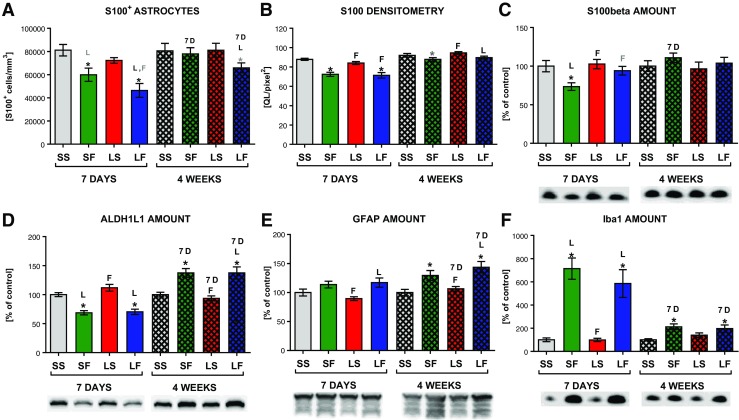
Stereological counting of astrocyte cell bodies (S100^+^) density in the SNc (**a**), S100 protein expression densitometry analysis in the SNc tissue sections (**b**), S100beta (**c**), ALDH1L1 (**d**), GFAP (**e**), and Iba1 (**f**) protein amount estimated by Western blot analysis in the whole SN homogenates with representative band pattern. Data are presented as the mean values ± SEM of **a** cell density, **b** optical density quant levels per area (QL/pixel^2^), and **c**–**f** percent of control from chemiluminescence arbitrary units/total protein. Two-way ANOVA and the LSD post hoc test were used, and Student’s *t* test was applied to compare time effect between groups with the same treatment. * vs sham + solvent; *L* vs 6-OHDA lesion + solvent; *F* vs FC + solvent; *7D* vs 7-day time-point; *marks* in *black* indicate *p* ≤ 0.05 and *marks* in *gray* 0.1 ≥ *p* > 0.05. Each group consisted of 6–12 animals for stereology, 6–11 for densitometry (9–12 sections per animal), and 5–6 for Western blot, in duplicate for GFAP and in triplicate for ALDH1L1, S100beta, and Iba1

Strongly depleted staining of both markers (GFAP and S100) in the SN after 7 days of FC infusion was visible in groups receiving the glial toxin alone and together with 6-OHDA (Fig. 4). A few remaining astrocytes at the site of FC infusion and around it showed changed morphology with stronger GFAP staining and thicker branches, indicating their activation (supplementary data Fig. 1). Stereological counting of S100^+^ cell bodies confirmed that 26 and 42.8% of astrocytes died in the SNc after 7 days of FC infusion in groups without or with 6-OHDA, respectively (S100^+^ cell density was 81118 ± 4958 in SS; 59941 ± 5714 in SF; 46397 ± 5988 in LF) (Fig. 5a). Densitometric analysis of tissue sections stained for S100 in the SNc showed the decreased optical density in respective groups, as well (Fig. 5b). The amount of this protein in the SN also decreased significantly after FC and non-significantly after FC and 6-OHDA treatment (Fig. 5c). In addition, the amount of another astrocyte-specific, yet functionally unrelated, protein ALDH1L1 [63] estimated using Western blot analysis in SN homogenates decreased significantly after 7 days of FC infusion to the same extent in both groups: without and with 6-OHDA injection (Fig. 5d).

While a smaller number of astrocytes and their changed morphology were clearly visible on tissue sections, GFAP amount estimated by Western blot analysis in the whole SN (pars compacta + reticulata) (Fig. 5e) was not reduced after 7 days of FC infusion, further indicating activation of the remaining and surrounding astrocytes manifested by their increased GFAP expression (Fig. 4 and supplementary data Fig. 1).

The effect of FC was still visible in tissue staining after 4 weeks but was much smaller (Fig. 4). More S100^+^ and GFAP^+^ astrocyte cells were visible in the SN region, suggesting regrowth of astrocytes. This was confirmed by stereological counting of astrocytes in the SNc (Fig. 5a). Their overall density returned to control levels in FC alone group (96.8% of control) but was still decreased, although not statistically significant, in 6-OHDA + FC group (81.7% of control) (density of S100^+^ cells 80592 ± 6379 in SS; 77977 ± 5291 in SF; 65847 ± 4338 in LF). Also, densitometric analysis indicated that the number of S100^+^cells returned to near-control levels (Fig. 5b) but S100 expression in FC alone group was still slightly lower. Control level of S100beta protein or increased amounts of both ALDH1L1 and GFAP protein estimated in Western blot analysis in both groups further indicate the reversal of the FC effect (Fig. 5c–e).

No macroscopic changes in the SNc were observed after injection of 6-OHDA alone into the MFB. Stereological counting of astrocyte cell bodies stained with S100 did not show any significant degenerative changes, and the amount of GFAP protein estimated by Western blot analysis did not change.

### Reversible Microglia Activation After FC Infusion

**Fig. 6:**
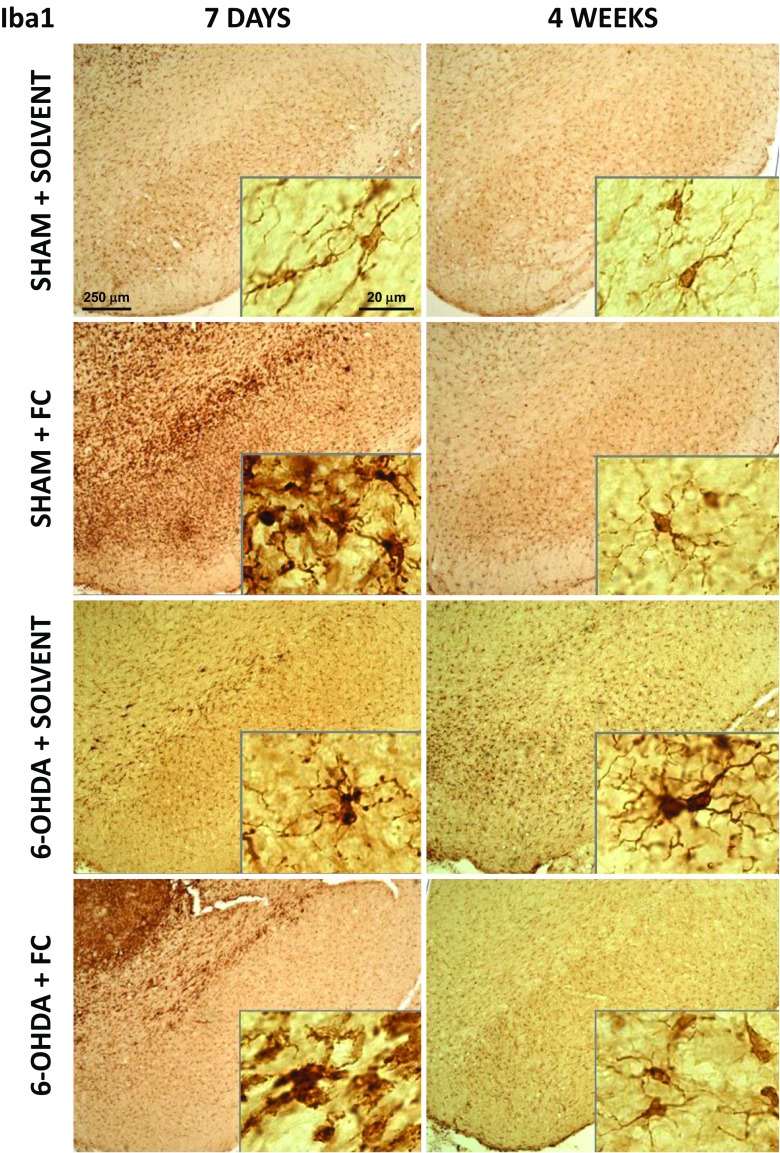
Distribution and morphology of Iba1^+^ immunostained microglia in the SNc tissue sections. *Scale bars* represent 250 and 20 μm, under ×5 and ×63 magnifications, respectively. Activation of microglia was visible after 7 days of FC infusion. This effect was reversed after 3 weeks of toxin withdrawal in both groups without and with 6-OHDA injection. Small activation of microglia due to dopaminergic neuron degeneration was also visible

In control sections examined both 7 days and 4 weeks after operation, microglial cells had long, thin, and branched processes. Infusion of FC into the SNc resulted in a massive activation of Iba1^+^ microglial cells (Fig. 6). After 7 days, they became much more strongly stained, and their processes became shorter and thicker with visible varicosities. In FC + 6-OHDA group, microglia were even more activated with enlarged cell bodies and almost no processes. Interestingly, in both cases, after additional 3 weeks after the end of FC infusion, microglial cells returned to the normal state.

After a single injection of 6-OHDA into the MFB causing the progressive dopaminergic neuron degeneration, a slight activation of microglia was also visible, manifested by a stronger Iba1^+^ staining of both cell bodies and processes (Fig. 6). Terminals were still long and branched but slightly thicker and with varicosities. This effect was detected after 7 days and continued also till the 4th week post operation.

Iba1 Western blot results showed the corresponding effect, with highly increased protein amount after FC in both groups without and with 6-OHDA after 7 days and diminished, yet still significantly higher than control amount after 4 weeks (Fig. 5f).

### Time-Dependent Changes in DA Levels, Metabolism and Turnover Due to Prolonged FC Infusion and Degeneration of Dopaminergic Neurons

Injection of 6-OHDA into the MFB resulted in a progressive decrease in DA and DOPAC tissue concentration in the STR (decrease by 74.6 and 93% after 1 and 4 weeks for DA and decrease by 58.6 and 82.2% after 1 and 4 weeks for DOPAC, respectively) and to a lesser degree in the SN (decreased DA by 37 and 79% after 1 and 4 weeks and DOPAC only after 4 weeks by 83.5%) (Table 2, supplementary data Fig. 2). Interestingly, selective, 6-OHDA-induced neuronal lesion significantly enhanced DA turnover rates in the STR (increase by 89.4 and 203.2% after 1 and 4 weeks). Selectivity of dopaminergic lesion was confirmed by lack of changes in serotonergic parameters (Table 2).

**Table 2:** HPLC analysis of DA, serotonin (5-HT), their metabolites, and turnover rates in the SN and STR

	7 days				4 weeks			
	SS	SF	LS	LF	SS	SF	LS	LF
STR								
DA	11941 ± 447	9067 ± 1000*^L^	3032 ± 484*^F^	966 ± 209*^LF^	10137 ± 687^7d^	11288 ± 532^7dL^	821 ± 226*^7dF^	2345 ± 424*^7dF^
DOPAC	934 ± 32	1071 ± 104^L^	387 ± 33*^F^	188 ± 36*^F^	794 ± 60	989 ± 84^L^	167 ± 42*^7dF^	330 ± 87*^F^
HVA	683 ± 71	872 ± 129^L^	275 ± 17*^F^	134 ± 25*^F^	613 ± 62	786 ± 70^L^	110 ± 16*^F^	196 ± 54*^F^
3-MT	367 ± 17	353 ± 17^L^	220 ± 9*^F^	70 ± 17*^LF^	350 ± 19	358 ± 8^L^	89 ± 23*^7dF^	145 ± 32*^7dLF^
DOPAC/DA	0.078 ± 0.002	0.119 ± 0.005	0.136 ± 0.015*	0.211 ± 0.023*^LF^	0.078 ± 0.002	0.087 ± 0.005^L^	0.221 ± 0.022*^7dF^	0.186 ± 0.035*^F^
HVA/DA	0.057 ± 0.005	0.094 ± 0.010	0.098 ± 0.012	0.152 ± 0.021*^LF^	0.060 ± 0.004	0.069 ± 0.005^L^	0.174 ± 0.032*^7dF^	0.086 ± 0.014^7dL^
3-MT/DA	0.031 ± 0.002	0.042 ± 0.004^L^	0.081 ± 0.014*^F^	0.074 ± 0.010*^F^	0.035 ± 0.001	0.032 ± 0.001^L^	0.110 ± 0.008*^7dF^	0.067 ± 0.011*^LF^
Turnover	0.167 ± 0.007	0.256 ± 0.014	0.315 ± 0.039*	0.438 ± 0.049*^LF^	0.173 ± 0.006	0.189 ± 0.008^L^	0.505 ± 0.055*^7dF^	0.339 ± 0.047*^LF^
5-HT	372 ± 41	333 ± 21	337 ± 31	411 ± 47	315 ± 16	356 ± 30	335 ± 46	345 ± 40
5-HIAA	419 ± 20	407 ± 18	411 ± 21	550 ± 38*^LF^	337 ± 24^7d^	404 ± 16	375 ± 29	412 ± 19*^7d^
5-HIAA/5-HT	1.17 ± 0.08	1.26 ± 0.08	1.24 ± 0.05	1.41 ± 0.15	1.08 ± 0.08	1.17 ± 0.08	1.18 ± 0.10	1.28 ± 0.14
SN								
DA	712 ± 42	688 ± 60^L^	448 ± 25*^LF^	713 ± 83	606 ± 51	622 ± 82^L^	147 ± 23*^7dF^	130 ± 40*^7dF^
DOPAC	114 ± 6	183 ± 19*^L^	93 ± 14^F^	190 ± 28*^L^	89 ± 8	117 ± 16^7dL^	19 ± 3*^7dL^	17 ± 6*^7dL^
HVA	50 ± 3	125 ± 17*^L^	40 ± 6^F^	161 ± 14*^LF^	35 ± 3	61 ± 10^7dL^	9 ± 1^F^	11 ± 1^7dF^
DOPAC/DA	0.162 ± 0.008	0.267 ± 0.013*	0.212 ± 0.036	0.279 ± 0.048*^L^	0.147 ± 0.007	0.187 ± 0.010^7d^	0.128 ± 0.010^7d^	0.133 ± 0.013^7d^
HVA/DA	0.072 ± 0.006	0.178 ± 0.017*^L^	0.093 ± 0.017^F^	0.246 ± 0.041*^LF^	0.058 ± 0.003	0.097 ± 0.011^7d^	0.063 ± 0.005	0.103 ± 0.021^7d^
Turnover	0.234 ± 0.012	0.418 ± 0.029*^L^	0.262 ± 0.028^F^	0.524 ± 0.085*^LF^	0.205 ± 0.009	0.284 ± 0.019^7d^	0.190 ± 0.011	0.235 ± 0.025^7d^
5-HT	569 ± 53	451 ± 28	528 ± 95	487 ± 62	631 ± 38	554 ± 44	588 ± 61	509 ± 105
5-HIAA	430 ± 30	484 ± 38	515 ± 59	545 ± 51*	331 ± 9	408 ± 35	334 ± 23^7d^	337 ± 36^7d^
5-HIAA/5-HT	0.775 ± 0.055	1.113 ± 0.125*	0.874 ± 0.196	1.165 ± 0.121*	0.531 ± 0.025	0.752 ± 0.076^7d^	0.575 ± 0.021	0.753 ± 0.143^7d^

Turnover rate in the STR was calculated as [DOPAC + HVA + 3-MT]/DA and in the SN as [DOPAC + HVA]/DA, because 3-MT was below detection limit in the SN. Data are presented as nanograms per milligram of tissue mean values ± SEM. Two-way ANOVA and the LSD post hoc test were used, and Student’s *t* test was applied to compare time effect between groups with the same treatment. Each group consisted of 4–9 animals

*p ≤ 0.05 vs sham + solvent; ^L^ *p* ≤ 0.05 vs 6-OHDA lesion + solvent; ^F^ *p* ≤ 0.05 vs FC + solvent; ^7d^ *p* ≤ 0.05 vs 7-day time-point

FC infusion into the SN caused only a temporary decrease in DA content by 24% after 7 days in the STR but not in the SN where DA metabolism and turnover rates were strongly enhanced (increase by 60.8% in DOPAC and by 79% in DOPAC/DA), masking the DA deficit. This effect in the SN returned to near-control values after 4 weeks and was not detected in the STR at this time-point (Table 2).

The combined treatment with 6-OHDA and FC depleted DA and decreased the level of its metabolite DOPAC in the STR in a very fast manner, already after 1 week (decrease in DA by 92% and in DOPAC by 80%), showing a small reversal of this effect after 4 weeks (decrease by 80% in DA and by 64.6% in DOPAC). At the same time, highly increased turnover rates ([DOPAC + HVA + 3MT]/DA increase by 163 and by 103.4% after 1 and 4 weeks, respectively) were observed in the STR. On the 7th day, this effect was higher than after 6-OHDA treatment alone and decreased after 4 weeks (Table 2, supplementary data Fig. 2).

Interestingly, despite fast neuronal degeneration in the SN after 6-OHDA + FC, there was no decrease in DA levels in the SN directly after 7 days. This was probably also due to highly increased DA metabolism and turnover (DOPAC 167% of control and DOPAC/DA 224.4% of control; [DOPAC + HVA]/DA 223.9% of control) (see Table 2, supplementary data Fig. 2). The decrease in DA and DOPAC level in the SN was detected only after 4 weeks (decrease by 79 and by 80.9% respectively), and was of the same magnitude as after 6-OHDA injection alone, after the enhanced turnover of DA returned almost to control levels.

Importantly, FC administered alone or together with 6-OHDA strongly enhanced DA metabolism and turnover in the SN. It is noteworthy that DA turnover rates never dropped below the control values, even after 6-OHDA-induced degeneration of dopaminergic neurons in the SN was accomplished, indicating highly increased production of DA per a single remaining neuron.

## Discussion

### Activation and Degeneration of Astrocytes

In contrast to neurons, little information is available on changes accompanying the degenerative process of astrocytes in vivo. The data presented here confirm that a local, chronic infusion of FC affects functioning of astrocytes, causing their activation and degeneration. We observed a decreased staining for both GFAP and S100 in tissue sections of the SN and reduced density of S100^+^ cell bodies as well as decreased amount of another astrocyte specific protein ALDH1L1, after 7 days of FC administration. The remaining astrocytes were strongly activated what was indicated by their morphology and increased GFAP expression. Furthermore, even though the lack of cells was clearly visible on tissue sections, there was no decrease in the amount of GFAP protein measured by Western blot in the SN. This corresponds with previous studies reporting that dysfunctional astrocytes are hypertrophic, overexpress GFAP [64–66], and become activated before apoptosis [67]. Therefore, since the number of the remaining activated astrocytes was smaller but their GFAP expression was increased, the overall result of Western blot analysis showed equal to normal amount of this protein in tissue samples. In addition, in our study, the stereological cell counting was performed in the SNc while Western blot analysis was carried out in the whole SN, containing both *pars compacta* and *reticulata*; thus, the effects from both these structures were combined. Previous studies also have shown that reactive astrocytes migrate to the lesion border extending their hypertrophied cellular processes to the injury site [68]. The analysis of S100beta protein amount in the SN indicates actual cell death of astrocytes.

### Neuronal Stress but Not Cell Death Induced by FC and Dysfunction of Astrocytes

We also showed here that chronic infusion of a low FC concentration (2 nmol/24 h, for 7 days) did not induce a significant neuronal cell death in the SN. No statistically significant changes were detected in cell counts after 4 weeks compared to sham-operated animals; thus, we conclude that no neuronal degeneration was induced by FC treatment alone. However, since we observed some temporary decrease in dopaminergic phenotype expression, FC treatment and astrocyte deficit had to stress neurons. It indicates that prolonged FC treatment puts neurons under stress but not necessarily kills them.

This is in agreement with the supportive role of astrocytes and metabolic coupling between neurons and astrocytes, proposed previously [28, 69]. Therefore, prolonged lack or impaired astrocyte functioning due to the FC-induced inhibition had to put also neurons under metabolic stress, although it did not kill them in the present experimental setting. FC has been shown previously to be predominantly taken up by glial cells through monocarboxylate transporter-1 and to inhibit the Krebs cycle enzyme aconitase, resulting in reduction of glial ATP production and overall metabolic stress [45–47, 70–73]. Similarly to our data, selectivity of FC towards glial cells, without neuronal cell death, was proven before in a dose range up to 2 nmol after acute intrastriatal injection [45, 70]. In the study by Zielke et al. [46] after infusion of FC at 100 μmol/l/1 h through a microdialysis probe (effective dose 1.8 nmol) into the hippocampus, no evidence of neuronal damage was detected. FC at concentrations of 5–100 μmol/l in vitro inhibited glial but not neuronal aconitase activity [47, 74]. Electrophysiological studies on hippocampal slices using fluoroacetate (FC precursor) doses as high as 10–20 mM showed a decreased synaptic transmission through blockade of glutamate uptake by astrocytes, but neuronal electrogenic membrane function was unaffected [25]. Compared with previous studies, the active dose administered here was much lower (0.083 nmol/μl/h) but on the other hand, it was administered constantly for 7 days and cumulative dose was 14 nmol. The effects of acute FC injection were described to be entirely reversible after 24 h [45, 70]; therefore, we infused FC in a constant manner, through osmotic minipumps at a very slow rate (0.5 μl/h), to obtain the chronic effect. Knowing that astrocytes can proliferate, we used the prolonged FC infusion instead of a single dose injection to induce stress for a significant time, before they were able to replenish their pool. The dose was chosen to obtain a moderate, yet still manageable effect. We wanted to avoid just preconditioning with too small dose. In a higher dose, FC could be toxic to neurons and the effect of astrocyte dysfunction would be unrecognizable. Moreover, FC in higher doses decreases seizure threshold and might induce epileptic states [75].

The described treatment allowed us to study the effects of small changes that are often not detected as disease itself but cumulate with other factors and manifest for example with aging, like in PD. The presented model can be also used to study pathology of other diseases accompanied by astrocyte dysfunction. Astrocytes contribute also to neurodegenerative processes seen in amyotrophic lateral sclerosis, Alzheimer’s and Huntington’s diseases, and in major neuropsychiatric disorders, like schizophrenia and depression, as well as in addictive disorders [33, 35, 76].

### Reversibility of the FC Effect

In contrast to neuronal cells, astrocytes keep their proliferative potential also in the adult brain. Emsley and Macklis [61] estimated the number of newly generated astroglia in the brain over a 7-day period at approximately 10%. Interestingly, we observed that 3 weeks after discontinuation of FC infusion, the GFAP and S100 staining-deprived area on tissue sections was smaller and astrocyte cell body count returned to the control levels, while ALDH1L1 and GFAP amounts increased. All these data indicate that FC treatment was reversible and the astrocyte pool was replenished in the SN. This makes the animal model presented here very useful for future studies on prospective regenerative or neuroprotective potential of astrocytes.

### Astrocytes in the SN

Previous studies investigating astrocyte degeneration or dysfunction usually demonstrated changes in regions other than the SN [48–50, 52, 77]. Our study for the first time showed chronic astrocyte inhibition in the SN. The only other study by Rodriguez-Diaz et al. [78] used acute FC injection into the SN through a microdialysis probe. They showed that the blockade of astrocyte function inhibited their glutamate uptake from synapses and significantly increased extracellular glutamate concentration in the rat SN 2 h after the infusion but they never studied the effect of FC on dopaminergic neurons. The studies presented here show for the first time the effect of prolonged astrocyte dysfunction in the SN. It has strong implications for PD pathogenesis and, although it is an animal model, it further proves that impaired astrocyte functioning in the human midbrain could be the underlying cause of PD due to acceleration of naturally occurring neuronal cell death caused by aging or increased vulnerability of neurons to insults during lifetime.

### The Role of Dysfunctional Astrocytes in Degeneration of Dopaminergic Neurons

The main finding of this study is that FC caused prolonged astrocyte dysfunction and accelerated neuronal degeneration in the SN induced by the selective dopaminergic toxin 6-OHDA. Stereological analysis in the SNc indicated decreased TH^+^ neuron density already 7 days after combined treatment with 6-OHDA and FC, when the effect of 6-OHDA alone was still only partial. Degeneration of neurons in 6-OHDA group was progressive and after additional 3 weeks reached the same level as after treatment combined with FC. The toxicity induced by both agents was not enhanced in the 4th week probably due to a small FC dose and reversibility of its effect after discontinuation. If the inhibition was longer or applied before 6-OHDA, much higher neurodegeneration could be expected. The question arises about the threshold of astrocytic dysfunction that would be required to induce neuronal cell death.

Interestingly, there are no previous studies on dysfunction of astrocytes in the SN in vivo or its influence on dopaminergic neuron vulnerability. Similar to our results, the in vitro study by McNaught and Jenner [79] did indicate that glial dysfunction might cause neuronal death or render neurons susceptible to toxic insults. They showed that 6-OHDA- or MPP^+^-induced neuronal death was enhanced in mesencephalic cultures previously cultured with lipopolysaccharide-activated or glutathione-depleted astrocytes. Those results let them formulate hypothesis that activated or dysfunctional astrocytes might make neurons vulnerable by a mechanism involving the release of free radicals and glutamate. Indeed, multiple studies have shown that transition of astrocytes from resting to the reactive state is associated with secretion of molecules, such as cytokines, eicosanoids, ROS, nitric oxide, and excitatory amino acids (see [27] for review). Taking into account also energetic support of astrocytes and their actual death in our study as well as acceleration but not direct induction of neuronal degeneration by FC treatment, we suggest that prolonged astrocyte dysfunction probably decreases reserve energy capacity of the dopaminergic system. Our results implicate that without astrocytic support, dopaminergic neurons in the SNc are stressed but able to survive for some time if there are no additional insults or environmental toxicants. Under normal conditions, no dysfunction is observed but in reaction to stress or insult, the safety threshold is exceeded, energy deficiency occurs, and neurons could undergo exhaustion and in consequence degenerate. Also, withdrawal of neuroprotective and antioxidant support can be detrimental for longer neuronal functioning in stressful situations when astrocyte function is impaired.

### Microglia Activation Due to FC and Astrocyte Stress

We stained tissue sections for the microglial marker Iba1 (Fig. 6), and their activation was clearly visible after FC treatment and diminished with time after FC withdrawal. Iba1 protein amount in the SN estimated by Western blot analysis further corroborates these findings. In this study, we cannot discriminate which cell type was affected first astrocytes or microglia. Previous studies with FC indicated generally the effect on glia [70, 72]. Although we documented S100^+^ astrocyte cell death, the microglial markers strongly increased; therefore, FC probably did not induce their death.

Activated astrocytes release certain molecules, such as chemokines and cytokines (ICAM-1 and IL-6), which act as stimulators of microglia migration [35] and stressed astrocytes can activate microglia [21, 80]. In this study, it is not possible to discriminate whether microglia activation was mediated by astrocyte stress or due to a direct FC toxicity. Similar results were obtained by Reenilä et al. [81] after FC injection into the STR. The FC effect on astrocytes and microglia is inseparable. It is not possible to induce cellular dysfunction and massive degeneration of astrocytes without activating the microglia, the intrinsic function of which is to react to stress and clear out the cellular debris [19]. Regrowth of astrocytes and diminishing of microglia activation corresponded in time. Functions of astrocytes and microglia are very much interrelated and they mutually regulate their activation state [19, 20]. The majority of studies described primarily activated microglia cells which than promoted astrocytic activation [82]. On the other hand, activated astrocytes can facilitate activation of distant microglia, as well as inhibit microglial activity [21]. Therefore, the other sequence of activation is possible, especially when primary trigger is not a viral or bacterial pathogen or brain injury.

Literature data often indicated the inflammation-driven or lipopolysaccharide-induced astrocyte activation and neuronal degeneration caused by activated microglia [80, 82]. However, we did not see a significant neuronal cell death after FC alone when microglia was activated or any enhancement of neuronal degeneration after combined treatment with FC and 6-OHDA in our study. Therefore, it is not the activated microglial cells that induced dopaminergic neurodegeneration here. They could be the cause of temporary neuronal stress described above in the FC, 7-day group. On the other hand, we did describe a faster degeneration after FC + 6-OHDA and activated microglia could participate in this process, for example in more efficient removal of cell bodies.

### FC Influence on DA Metabolism

FC infusion into the SN for 7 days dramatically enhanced DA metabolism and turnover in this structure. This effect was much smaller 3 weeks after FC discontinuation but still visible, correlating with astrocyte regrowth. Some studies have shown that astrocytes control neuronal excitability and can modulate synaptic transmission by inhibiting it, for example, through release of GABA [24]; thus, dysfunction and death of astrocytes can possibly disinhibit local neuronal network (see [27] for review).

The other explanation is that since a part of astrocytes was defective or degenerated 7 days post operation, thus a signal for enhancement of DA turnover probably came from the remaining activated astrocytes or non-astrocytic sources. In addition, since the same effect was observed in groups in which both astrocytic and neuronal deaths were observed, this enhancement trigger was probably of non-neuronal origin, which thus possibly came from microglia. The increased DA turnover in the SN after 7 days and its normalization after 4 weeks correspond not only to the astrocyte replenishment but also to microglia activation and their later return to near control state.

In line with our observation, Reenilä et al. [81] showed that 3 days after FC infusion into the STR, the activities of DA metabolizing enzymes were significantly changed. MAO-B activity, located predominantly in astroglia, but not in microglia, decreased, corresponding to astrocyte dysfunction and damage, whereas COMT activity was increased and co-localized mostly with microglial cells, but not with neurons or astrocytes (see [83] for comparison). Catecholamine metabolism is a complex process, and both mentioned enzymes are needed simultaneously to break down DA molecules in both glial cells as well as in neurons, and as the numbers of all those cells changed, we did not see clear cut changes in particular metabolites but rather an overall strong increase in DA turnover.

The enhanced DA turnover was observed also in STR in this study along with overcompensation of motor behavior after 6-OHDA and a much smaller rebound effect visible as the lack of motor deficit in FC + 6-OHDA group on the 6th day after operation. It indicates that DA release in the STR, not in the SN, is responsible for locomotor output regulation in the dopaminergic system. Interestingly, after impairment of both astrocytes and neurons on the 6th day, although neuronal degeneration was completed, the DA turnover was still strongly enhanced, thus rescuing the behavioral outcome. After 4 weeks, DA turnover in the STR was slightly lower although still enhanced in both lesioned groups (LS and LF) but in animals with previous astrocyte dysfunction, this effect was diminishing, indicating an important role of proper astrocyte functioning in long-term compensation.

### Dopaminergic Neuron Degeneration Process

Lesioning of dopaminergic neurons alone increased DA turnover in the STR. Such effect in animal models was observed at the early degeneration stages when a small number of neurons were affected [53, 84, 85]. Studies in primates and humans confirmed this observation also before the threshold level of degeneration required for parkinsonian symptoms was reached and suggested that it could likely serve as an early compensatory mechanism [11, 12]. Our data correspond with increased DA turnover as a marker of the preclinical stage of PD.

Furthermore, a slight activation of microglia in the SNc after a single 6-OHDA injection into the MFB correlated with progressive degeneration of dopaminergic neuron bodies.

### Blockade of Behavioral Compensation

The important aspect to consider is the blockade of the compensatory potential of dopaminergic system induced by dysfunction of astrocytes. In the case of small 6-OHDA lesion, the dopaminergic system was able to upregulate its remaining and postsynaptic neuron function in order to maintain motor activity of animals [9, 44, 53, 86] but when astrocytic support had been withdrawn, this compensation ability was lost. This effect was observed both as the loss of overcompensation in locomotor activity 6 days after operation and as still lowered parameters of behavior after 4 weeks. Surprisingly, the effect of diminished compensation was long-lasting, even after astrocytes regenerated. It also did not depend on degeneration extent since at the end of the experiment no difference in dopaminergic neuron density was observed between 6-OHDA with and without FC groups. This indicates that astrocytic support is essential for compensation of moderate and early neuronal deficits.

Four weeks after lesioning, the increased DA turnover rate in the STR was further enhanced in neuronal lesion group, where degeneration was progressive and behavioral compensation active. At the same time, after induction of combined astroglial and neuronal deficits, the initially enhanced DA turnover rate decreased with time in the STR. In this group, neuronal degeneration was of similar magnitude to 6-OHDA alone, although completed much earlier, resulting in the lack of behavioral compensation at 4 weeks. Since the neuronal lesion size was the same in both groups, these results suggest that the compensatory capability of dopaminergic neuronal system was depleted with time after completion of neuronal degeneration. This indicates emptying some reserve capabilities of the remaining cells and corresponds with still disturbed ability of astrocytes to support neurons.

Extinguishing of the microglia activation coincided with improvement of behavioral functions after FC alone. Disturbed compensatory potential after lesioning was observed in the group where microglial cells were activated during FC infusion. Their pro-inflammatory action could be another factor decreasing later the compensatory potential of remaining dopaminergic neurons. The exact role of microglia activation in the process of compensation of dopaminergic system functioning is an interesting question occurring from our study.

We postulate that early dysfunction of astrocytes in PD [40, 87] could be also a potential triggering factor of the dangerous long-term microglia activation in this disease. A better understanding of the crosstalk between activation states of microglia and astrocytes would be helpful to elucidate the role of glial cells in many pathological conditions.

## Conclusions

The present study shows that chronic infusion of a low FC concentration induced astrocyte dysfunction and degeneration as well as concurrent microglia activation, without causing neuronal cell death in the SN. This effect was reversible and astrocytes replenished their pool in the SN. This model is a good tool for studying small changes that can contribute to the neurodegenerative processes progressing with aging, like in PD. As presented here, new animal model of prolonged astrocyte dysfunction and microglia activation can be also used in studies relevant to a broad spectrum of central nervous system diseases and neuron–astrocyte–microglia interactions in vivo. This is the first study showing chronic astrocyte inhibition in the SN which proves that prolonged astrocyte dysfunction and microglia activation accelerate dopaminergic neuron degeneration induced by the selective dopaminergic toxin 6-OHDA. We also show that astrocytic support is essential for compensation of moderate neuronal deficits, which is especially interesting in respect to studying preclinical stages of PD. It implicates that astrocyte pathology could precede neuronal damage in early PD and interfere with endogenous protection. The impaired astrocyte functioning probably decreases reserve energy capacity in the dopaminergic system, lowering neuronal resistance threshold to stress and environmental insults and diminishing compensatory capability of the system.

Proliferative capacity of astrocytes gives an opportunity to implement protective therapies in the future. Pharmacotherapies enhancing astrocyte function as a way to “support the supporters” could become a new treatment perspective.

## Electronic supplementary material

ESM 1 (PPTX 9945 kb)

## Abbreviations

6-OHDA: 6-Hydroxydopamine
DA: Dopamine
FC: Fluorocitrate
GFAP: Glial fibrillary acidic protein
MFB: Medial forebrain bundle
PD: Parkinson’s disease
ROS: Reactive oxygen species
SN: Substantia nigra
SNc: Substantia nigra pars compacta
STR: Striatum
TH: Tyrosine hydroxylase
