# Supplementary material for: Prolonged Dysfunction of Astrocytes and Activation of Microglia Accelerate Degeneration of Dopaminergic Neurons in the Rat Substantia Nigra and Block Compensation of Early Motor Dysfunction Induced by 6-OHDA
Source: Mol Neurobiol. 2017 May 2;55(4):3049–66. doi: 10.1007/s12035-017-0529-z (PMC5842510; doi:10.1007/s12035-017-0529-z)
Supplement: Supplementary file 1 — (PPTX 9945 kb) [file 12035_2017_529_MOESM1_ESM.pptx]

## Slide 1
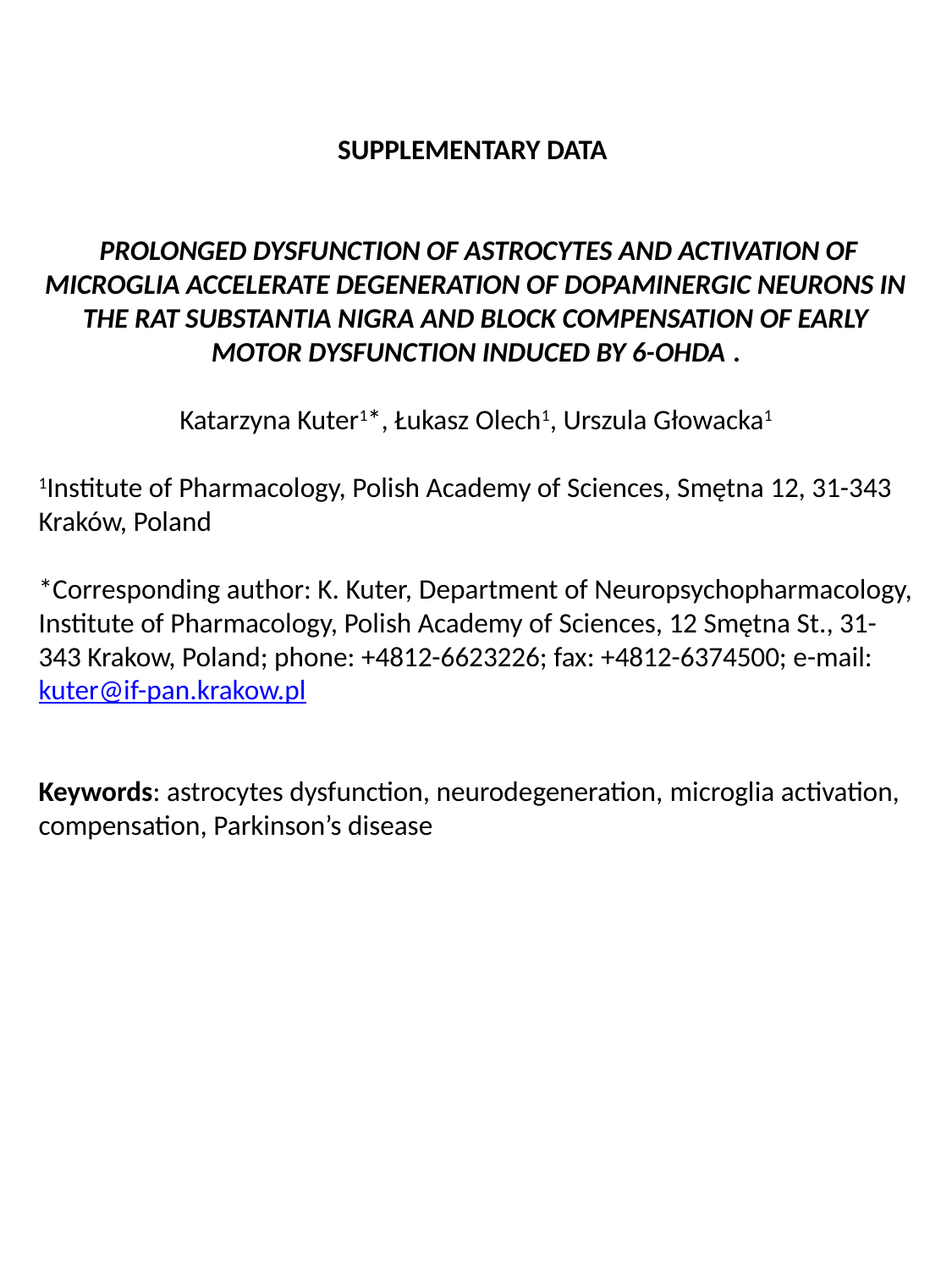

SUPPLEMENTARY DATA
 PROLONGED DYSFUNCTION OF ASTROCYTES AND ACTIVATION OF MICROGLIA ACCELERATE DEGENERATION OF DOPAMINERGIC NEURONS IN THE RAT SUBSTANTIA NIGRA AND BLOCK COMPENSATION OF EARLY MOTOR DYSFUNCTION INDUCED BY 6-OHDA .
Katarzyna Kuter1*, Łukasz Olech1, Urszula Głowacka1
1Institute of Pharmacology, Polish Academy of Sciences, Smętna 12, 31-343 Kraków, Poland
*Corresponding author: K. Kuter, Department of Neuropsychopharmacology, Institute of Pharmacology, Polish Academy of Sciences, 12 Smętna St., 31-343 Krakow, Poland; phone: +4812-6623226; fax: +4812-6374500; e-mail: kuter@if-pan.krakow.pl
Keywords: astrocytes dysfunction, neurodegeneration, microglia activation, compensation, Parkinson’s disease

## Slide 2
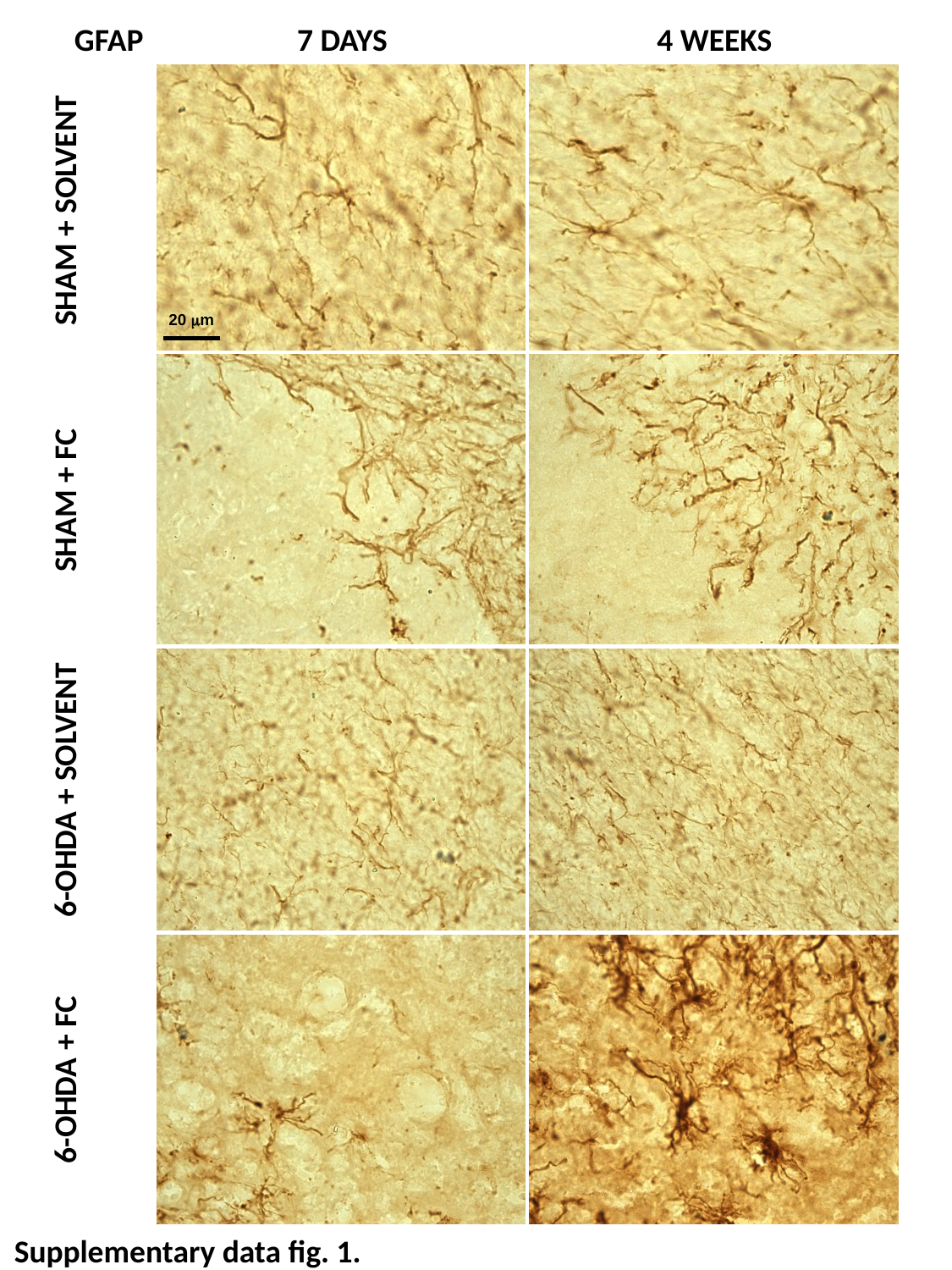

| GFAP | 7 DAYS | 4 WEEKS |
| --- | --- | --- |
| SHAM + SOLVENT | | |
| SHAM + FC | | |
| 6-OHDA + SOLVENT | | |
| 6-OHDA + FC | | |
20 μm
Supplementary data fig. 1.

## Slide 3
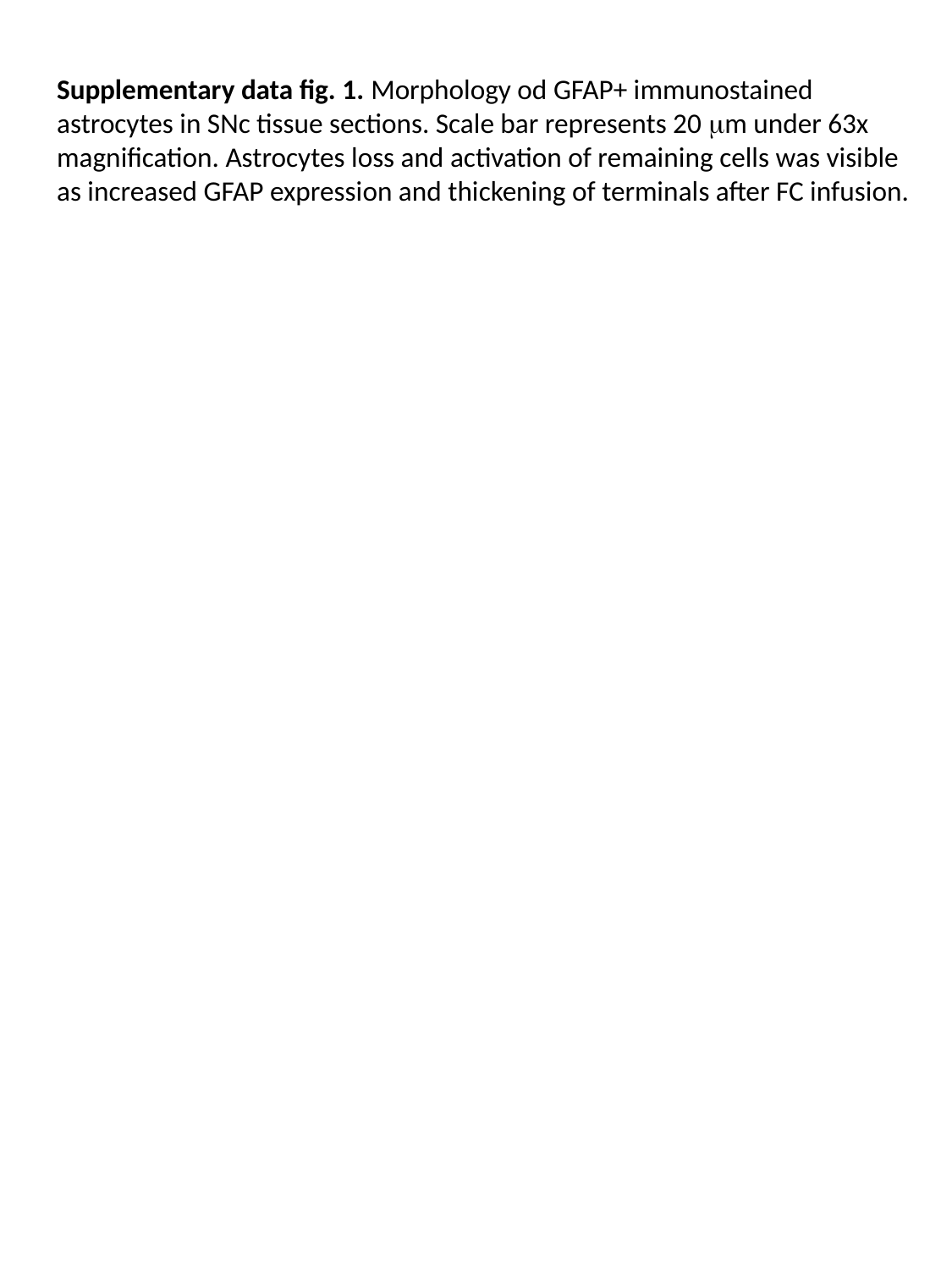

Supplementary data fig. 1. Morphology od GFAP+ immunostained astrocytes in SNc tissue sections. Scale bar represents 20 μm under 63x magnification. Astrocytes loss and activation of remaining cells was visible as increased GFAP expression and thickening of terminals after FC infusion.

## Slide 4
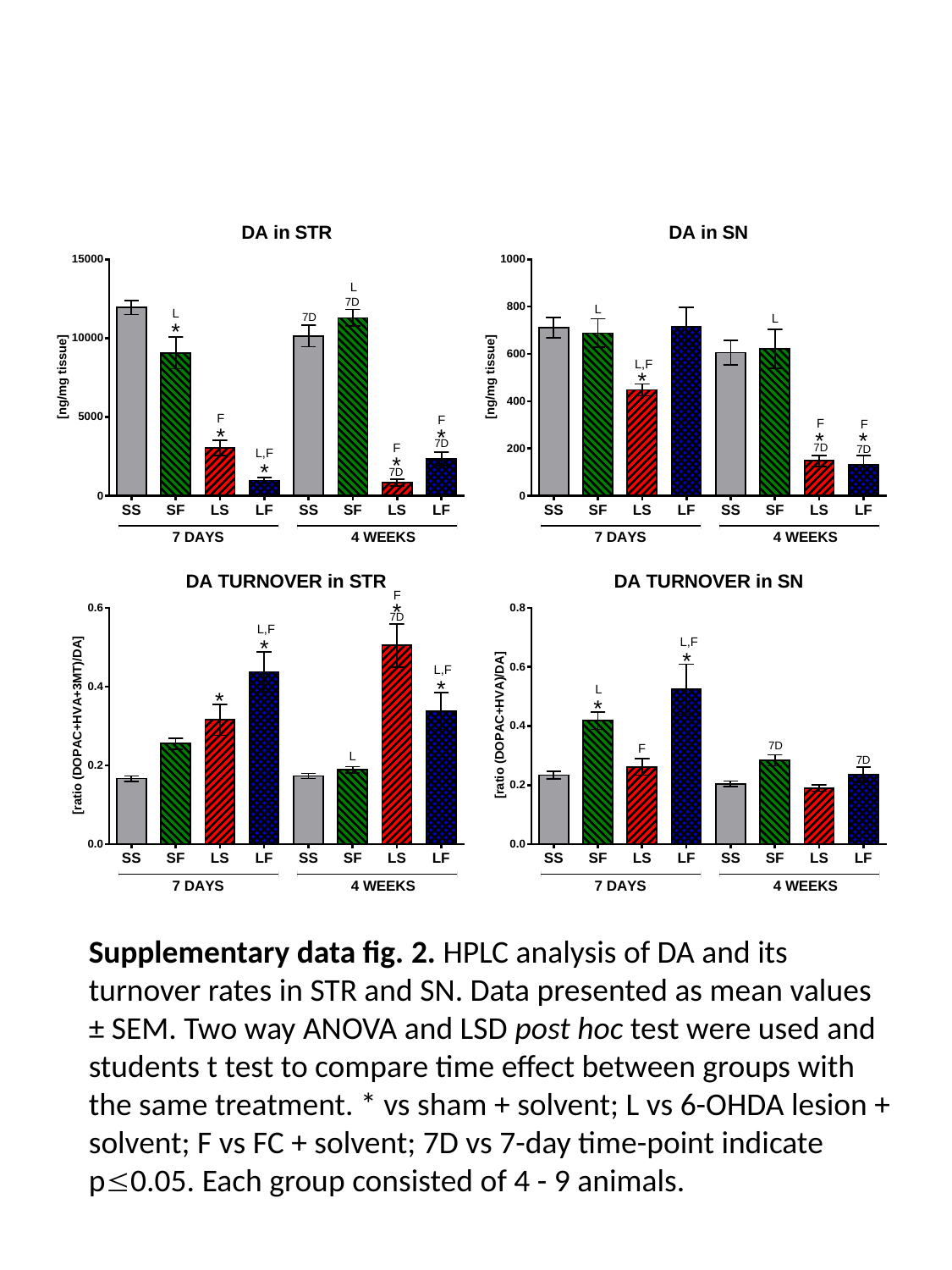

Supplementary data fig. 2. HPLC analysis of DA and its turnover rates in STR and SN. Data presented as mean values ± SEM. Two way ANOVA and LSD post hoc test were used and students t test to compare time effect between groups with the same treatment. * vs sham + solvent; L vs 6-OHDA lesion + solvent; F vs FC + solvent; 7D vs 7-day time-point indicate p≤0.05. Each group consisted of 4 - 9 animals.
